# Case Report: An extremely rare case of double extralobar pulmonary sequestration with anomalous supplying arteries originating from the abdominal aorta in the left thoracic cavity

**DOI:** 10.3389/fped.2022.926942

**Published:** 2022-07-22

**Authors:** Longfei Lv, Yunpeng Zhai, Huashan Zhao, Rui Guo, Hongxiu Xu, Shisong Zhang

**Affiliations:** ^1^Department of Thoracic and Oncological Surgery, Children’s Hospital Affiliated to Shandong University, Jinan, China; ^2^Department of Thoracic and Oncological Surgery, Jinan Children’s Hospital, Jinan, China

**Keywords:** pulmonary sequestration, extralobar sequestration, intralobar sequestration, thoracoscopic surgery, congenital lung malformation

## Abstract

To the best of our knowledge, double or multiple extralobar pulmonary sequestrations (PSs) with anomalous arterial supply in the ipsilateral thoracic cavity have rarely been reported before. PS can be divided into two types: intralobar sequestration (ILS) and extralobar sequestration (ELS). We encountered a 5-month-old infant with double ELS in the left thoracic cavity that was incidentally detected during thoracoscopic surgery. Surgical exploration revealed two separate, well-circumscribed abnormal masses in the left thoracic cavity, and the patient was successfully treated using thoracoscopic surgery. Postoperative pathology confirmed that both masses were PS tissues. Accurate preoperative diagnosis using CT alone may be inadequate in this type of case. Therefore, thoracoscopy may be more suitable for diagnosing and treating unusual ELS.

## Introduction

Pulmonary sequestration (PS), also known as accessory lung, is a lung malformation characterized by a mass of non-functioning lung tissue without connection to the normal tracheobronchial tree (1). In embryology, PS grows from an accessory lung bud that develops from the ventral aspect of the primitive foregut (2). However, double or multiple PS is extremely rare. There are several published case reports of bilateral (3, 4) and single PS with other ipsilateral thoracic malformations (5, 6) worldwide. However, ipsilateral intrathoracic double or multiple extralobar sequestrations (ELS) have never been reported. Here, we report a unique case of a 5-month-old child with intrathoracic double ELS incidentally found in the left thoracic cavity during thoracoscopic surgery. All masses were completely resected, and ELS was histologically confirmed in each mass postoperatively.

## Case description

**FIGURE 1:**
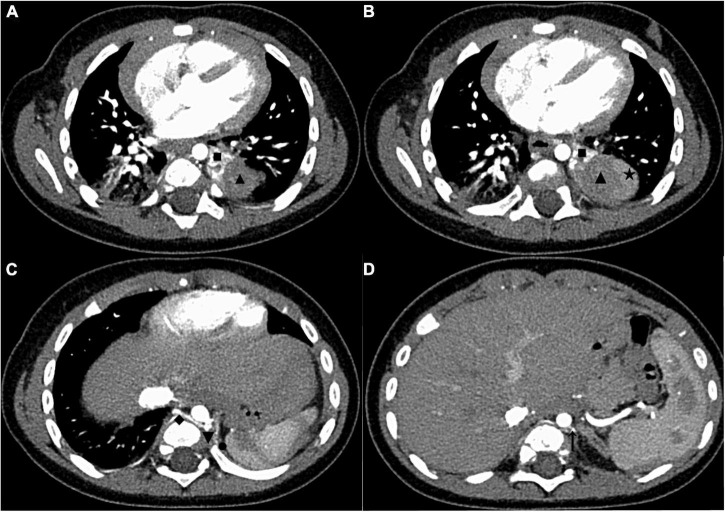
The enhanced chest CT of the case before surgery. **(A)** CT images in the AP showed two areas with different enhancement degrees in one mass, and the upper pole of the mass was adjacent to the inferior lobe of the left lung. **(B)** CT images in the AP showed that the largest cross-section of the mass was close to the upper pole of the spleen. **(C)** CT images in the AP showed the origin of the upper group of anomalous vessels (referring back to this image postoperatively, we found that it showed the origin of the anomalous supplying arteries and a draining vein of the upper ELS). **(D)** CT images in the AP showed the origin of the inferior group of anomalous vessels (referring back to this image postoperatively, we found that it showed the origin of an anomalous supplying artery of the inferior ELS). The draining vein of the inferior lesion cannot be distinguished. CT, computed tomography; AP, arterial phase; ELS, extralobar sequestration. ◼: upper ELS; ▲: inferior ELS; ★: spleen; ◆: draining vein of the upper ELS; ▼: the origin of anomalous supplying arteries of the upper ELS; ⚫: anomalous vessels in the left diaphragm connecting to the inferior ELS; ↑: the origin of an anomalous supplying artery of the inferior ELS.

The patient was a 5-month-old male infant diagnosed with ELS at the 22nd week of pregnancy. Ultrasound revealed a lesion adjacent to the inferior lobe of the left lung with a size of about 3 cm x 2.5 cm × 2 cm. Subsequent follow-up ultrasonography showed that the lesion volume had gradually increased. The child was born smoothly through vaginal delivery without any respiratory abnormalities such as respiratory distress. Since birth, the child developed normally and had no respiratory symptoms. The patient’s mother was very anxious about the risk of infection or torsion of the lesion since birth. The reason for this admission was that the infant’s mother required further imaging evaluation and surgical resection of the lesion. After admission, an enhanced chest CT revealed a clearly defined mass below the inferior lobe of the left lung with a size of 3.8 cm × 3.5 cm × 2.8 cm (Figures 1A,B). On the CT images below the lesion level, two groups of anomalous vessels with varying thickness were seen to originate from the abdominal aorta at different CT levels and ascend to connect to the mass. In addition, an anomalous vessel was seen to connect to the azygos vein at the same CT level in the upper group of anomalous arteries (Figures 1C,D). No abnormalities were observed on electrocardiography or echocardiography after admission.

**FIGURE 2:**
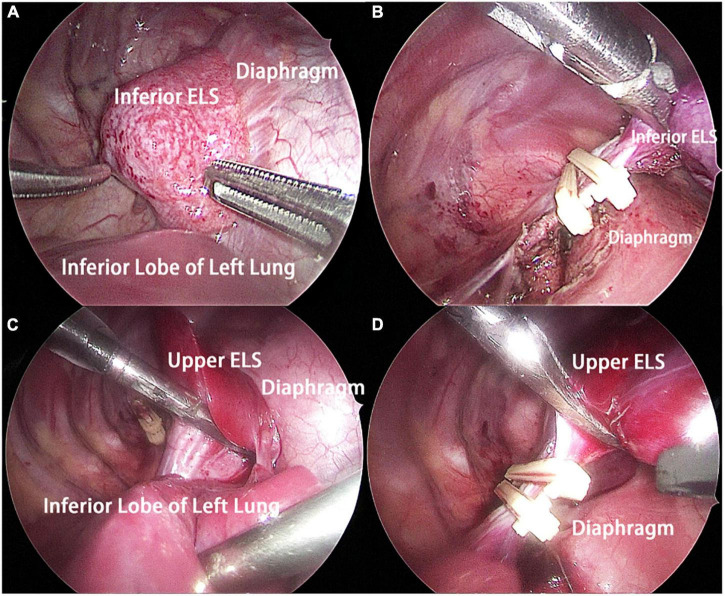
Intraoperative findings. **(A)** The inferior ELS was adjacent to the left diaphragm, and the base of the inferior ELS was closely related to the left diaphragm. **(B)** The inferior ELS was resected after double ligation of the anomalous vessels using two absorbable vessel clamps. **(C)** The upper ELS was adjacent to the left lower pulmonary ligament. Four anomalous vessels connecting to the upper ELS could be seen. **(D)** The upper ELS was resected after double ligation of the anomalous vessels using two absorbable vessel clamps. ELS, extralobar sequestration.

**FIGURE 3:**
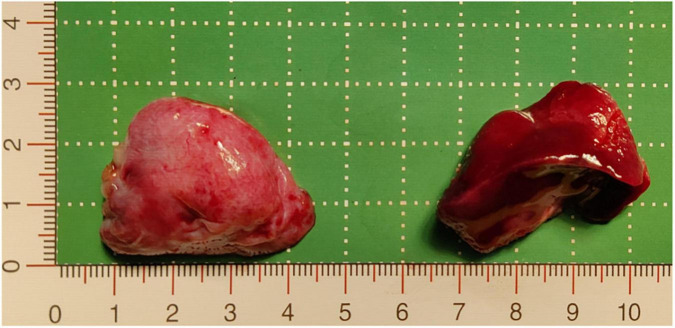
Resected pathological tissue. The pink tissue on the left of the image is the inferior ELS, which was approximately 3.7 cm × 2.7 cm × 2 cm, and the dark red tissue on the right of the image is the upper ELS, which was approximately 3.8 cm × 2.9 cm × 2.5 cm. ELS, extralobar sequestration.

**FIGURE 4:**
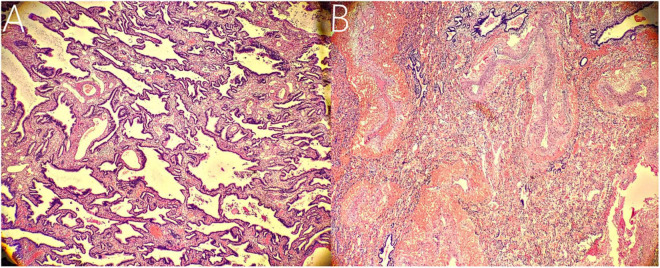
Histopathological findings. **(A)** Histopathological imaging of the inferior ELS. The tissue is mainly composed of abnormal airway epithelium, which contains fewer arterial and venous components with smaller diameters (hematoxylin and eosin stain, × 10). **(B)** Histopathological imaging of the upper ELS. The tissue is characterized by numerous arterial and venous components with larger diameters (hematoxylin and eosin stain, × 10).

The patient underwent thoracoscopic surgery on the 2nd day of admission. After general anesthesia, the patient was placed in the right lateral position. Surgery was performed under artificial pneumothorax by thoracoscopy using a 10 mm trocar and double 5 mm trocars *via* intercostal incisions. We unexpectedly found two separate and well-defined masses in the left thoracic cavity, whose colors were inconsistent. We named these two masses the inferior ELS and the upper ELS. The surface of the inferior ELS was pink and was covered with tortuous blood vessels. The base of the inferior ELS was closely related to the left diaphragm (Figure 2A). Incising part of the diaphragmatic fascia that was closely adhered to inferior ELS after sufficient dissociation. Several thin vessels appeared connected to the upper ELS in the medial basal tissue. The inferior ELS was resected after double ligation of the anomalous vessels using two absorbable vessel clamps (Figure 2B). The dark red upper ELS was adjacent to the left lower pulmonary ligament. Four anomalous vessels were observed in the connective tissue attached to the medial side of the upper ELS. The three upper anomalous vessels originated from a common vessel trunk (Figure 2C). The upper ELS was resected using the same method as the inferior ELS (Figure 2D). The operation took approximately 40 min, with approximately 5 ml of intraoperative bleeding. The two resected lesions were completely removed from the thoracic cavity and were diagnosed as PS through pathological examination (Figures 3, 4).

## Discussion

To our knowledge, this is the first report of a child with double ELS with anomalous arterial supply in the left thoracic cavity. Before this reported patient, the cases of rare PS mainly included the following types: variation of the source of arterial supply (7, 8), variation of venous drainage (9), variation of anatomical position (10), bilateral PS (11), and PS connecting with the digestive tract (12). A significant number of these patients were incidentally confirmed to have mutations during surgery. Similarly, our patient was conventionally diagnosed with single ELS preoperatively, in which the double masses were detected during thoracoscopic surgery.

The incidence of PS is approximately 0.1-6.4%, with ELS accounting for approximately 15-25% of these cases, which is relatively rare (13). ELS covered by its own pleura usually derives its blood supply from one or more abnormal independent arteries from the thoracic or abdominal aorta (4). ELS is usually located in the left thoracic cavity and between the lower lobe of the lung and the diaphragm (10). With the development of ultrasound equipment and technological experience, most fetal PS can be identified during pregnancy, and the location of lung lesions can also be roughly identified (14). In this case, the child was diagnosed with single ELS on the left side at the 22nd week of pregnancy using ultrasound. No significant change in lesion volume was observed until birth. This indicates that ultrasound may not be adequate for diagnosing variant ELS. Therefore, further examination after birth is needed to guide diagnosis and treatment.

The following imaging methods are used for diagnosing PS: ultrasonography, chest radiography, CT, MRI, and angiography. Chest radiography can provide an initial diagnostic clue for PS, especially in patients with local lesion infections. Angiography has been used for diagnosis in the past, especially for identifying feeding vessels; however, the recommended gold standard for diagnosing PS is now CT or MRI (13). CT angiography is the most commonly used method and can clearly show the origin of abnormal vessels supplying the lesion. However, in this case, CT angiography did not appear to be perfect for the definitive preoperative diagnosis of the two masses. When reading the CT images shown in Figures 1A,B before surgery, our first response was that there were two areas with different degrees of enhancement in one mass rather than two separate masses in the left thoracic cavity. Only when we looked back at the CT images of this patient combined with a surgery video postoperatively did we realize that these two areas were the upper and lower ELS. Moreover, the upper group of anomalous vessels in Figure 1C ascends to connect with the upper ELS. The inferior group of anomalous vessels in Figure 1D ascends to connect with the inferior ELS. CT images clearly showed that the draining vein of the upper ELS was injected into the azygos vein. However, the draining vein of the inferior ELS could not be distinguished on CT images. By directly observing the two pathological tissues seen in Figure 3, we can see that the inferior ELS is less vascularized than the upper ELS. The pathological images in Figure 4 also suggest that the vascular component of the inferior ELS is smaller than that of the upper ELS. In addition, the histopathologic image of the upper ELS was filled with red blood cells, which suggested signs of hemorrhage. Therefore, pathology further confirms a more significant enhancement degree in the upper ELS than in the lower ELS in the CT images. Studies have shown that MRI can provide a more detailed view of blood vessels and can find small PS that are not readily apparent in other imaging modalities (15). In conclusion, MRI may be necessary for the preoperative diagnosis of PS with variants in children.

The most appropriate management for ELS is controversial because some studies demonstrated that ELS may remain asymptomatic throughout life and may even disappear (16, 17). A significant number of patients with ELS have potential infection risks, with the infection rate varying from 16 to 31% (18). Additionally, a small number of patients with ELS are prone to torsion necrosis. Therefore, some scholars suggested active surgical treatment for all types of ELS (19). The upper ELS of this patient was found to be particularly prone to intraoperative torsion. Thoracoscopy achieved an accurate diagnosis in this patient and conveniently completed resection of the lesion, eliminating the risk of torsion in the future. Although the patient may benefit from the operation, preoperative preparation was not sufficient in this case. This case again warns us that we should make more detailed and dedicated efforts to refine preoperative diagnoses instead of hasty surgery when we encounter ELS with unusual imaging findings.

In conclusion, we reported a case of double extralobar pulmonary sequestration with anomalous arterial supply originating from the abdominal aorta. For such patients, it is difficult to obtain accurate diagnoses using only preoperative CT. MRI may be a necessary option before surgery to better visualize anomalous vessels and detect their variants. Therefore, thoracoscopy may be considered because of its minimally invasive diagnostic and therapeutic roles. In this case, surgeons should be reminded of the importance of careful intraoperative exploration to avoid omissive excisions when dealing with patients diagnosed with ELS preoperatively.

## Patient’s caretaker perspective

The mother of the patient: “I am very happy now. I am very surprised that there are two lesions in my baby’s chest and thank the doctors for dispelling my concerns.”

## Data availability statement

The original contributions presented in this study are included in the article/supplementary material, further inquiries can be directed to the corresponding author.

## Ethics statement

The studies involving human participants were reviewed and approved by the Ethics Committee of Children’s Hospital Affiliated to Shandong University and Jinan Children’s Hospital. Written informed consent to participate in this study was provided by the participant’s legal guardian/next of kin.

## Author contributions

RG and HX were responsible for the conception of the idea of the case report. YZ and HZ contributed to the data collection. LL prepared the first draft of the manuscript. SZ wrote a few sections of the report. All authors contributed to the revision and submission of the manuscript.

## Conflict of interest

The authors declare that the research was conducted in the absence of any commercial or financial relationships that could be construed as a potential conflict of interest.

## Publisher’s note

All claims expressed in this article are solely those of the authors and do not necessarily represent those of their affiliated organizations, or those of the publisher, the editors and the reviewers. Any product that may be evaluated in this article, or claim that may be made by its manufacturer, is not guaranteed or endorsed by the publisher.
